# Items

**Published:** 1878-06

**Authors:** 


Dr. Nathan Bozeman has been appointed to the position in the New York State Woman's Hospital held by the late Dr. E. R. Peaslee.—*Detroit Lancet*

---

The Medical College of the Pacific announces that it has adopted the plan of education at present followed by the University of Pennsylvania.—*Ibid.*

---

Up to January, 1877, Sir Henry Thompson had performed five hundred operations for stone in the bladder of adult males. The proportion of deaths in the entire number was one to eight and one half deaths.—*Ib.*

